# Hypertension in cancer patients treated with anti-angiogenic based regimens

**DOI:** 10.1186/s40959-015-0009-4

**Published:** 2015-12-07

**Authors:** Yishay Wasserstrum, Ran Kornowski, Pia Raanani, Avi Leader, Oren Pasvolsky, Zaza Iakobishvili

**Affiliations:** 1 Department of Cardiology, Rabin Medical Center, Petah Tikva, 49100 Israel; 2 Institute of Hematology, Davidoff Cancer Center, Rabin Medical Center, Petah Tikva, Israel; 3 Sackler School of Medicine, Sackler Faculty of Medicine, Tel Aviv University, Tel Aviv, Israel

**Keywords:** Tyrosine kinase inhibitor, Vascular endothelial growth factor, VEGF, VEGF signal pathway inhibitors, VSP inhibitors, Hypertension, Cardiotoxicity

## Abstract

New anti-cancer drugs that inhibit the vascular endothelial growth factor (VEGF) signaling pathway are highly effective in the treatment of solid tumors, however concerns remain regarding their cardiovascular safety. The most common side effect of VEGF signaling pathway (VSP) inhibition is the development of systemic hypertension. We review the incidence, possible mechanisms, significance and management of hypertension in patients treated with VSP inhibitors.

## Background

The importance of adequate diagnosis and management of hypertension in patients with underlying malignancy is well-known [1]. Poorly controlled hypertension influences cancer management, leading to temporary or complete cessation of life-saving therapies. Depending on the type and dose of treatment, systemic hypertension of new-onset is a common side effect of many anticancer agents, particularly the vascular endothelial growth factor (VEGF) signaling pathway (VSP) inhibitors. In this review article we discuss the evidence, proposed mechanisms and management of systemic hypertension secondary to VSP inhibition.

### Pathogenesis of hypertension secondary to VSP inhibition

Cancer cell growth and proliferation mostly depends on a blood supply, which is provided through angiogenesis [2]. Angiogenesis is controlled by many growth factors through their specific receptor tyrosine kinases and activation of multiple tyrosine kinase pathways. VEGF and its receptors (VEGFR) are one of the most important growth factor pathways and play major role in endothelial cell function [3]. VEGF inhibition has significantly advanced the treatment of cancers leading to prolonged survival in previously untreatable cancers. The VEGF pathway can be targeted at various levels in the signaling cascade, including the VEGF molecule (by monoclonal antibodies, eg, bevacizumab), its receptors (recombinant soluble VEGF receptor trap, Ziv-Aflibercept) or downstream signaling pathways (small molecule receptor and non-receptor tyrosine kinase inhibitors, e.g. sunitinib, sorafenib). While it was thought that adverse effects of anti-angiogenesis therapy would be minimal it has become evident that systemic hypertension is the commonest and most significant cardiovascular side effect [4].

VEGF binds three tyrosine kinase receptors (VEGFR-1 [Flt-1], VEGFR-2 [Flk-1/KDR], and VEGFR-3 [Flt-4]) [5]. VEGFR-1 and VEGFR-2 are expressed predominantly in endothelial cells, with VEGF-A binding to VEGFR-2 having the major biological effects [6]. Binding of VEGF to VEGFR-2 initiates a tyrosine kinase-signaling cascade that stimulates production of factors that induce vasodilation, cell proliferation/survival, migration, and differentiation into mature blood vessels.

The pathophysiological mechanisms that result in the development of hypertension during therapy with VSP inhibitors is unclear.

There are several proposed mechanisms for VSP inhibitor-associated hypertension: reduction in nitric oxide (NO) production, increased expression of pro-hypertensive agents such as endothelin-1 (ET-1), microvascular rarefaction, activation of renin-angiotensin system, oxidative stress, pressure-natriuresis system, mechanical properties of large vessels (arterial stiffness).

VEGF induces the release of NO and prostacyclin (PGI_2_) by endothelial cells [7]. Blocking VSP will therefore decrease the secretion of these vasodilators and elevate systemic vascular resistance with a subsequent increase in blood pressure [8]. Experimental data that antiangiogenic drugs decrease NO bioavailability is contradictory. VEGF inhibition in humans is associated with decreased serum levels of NO metabolites [9] while no difference in flow-mediated dilation, a surrogate for NO bioavailability, is observed.

An alternative mechanism is that hypertension results from increased levels of the potent vasoconstrictor ET-1. Sunitinib-induced hypertension is partially abolished by ET_A_ and ET_B_ receptor antagonist macitentan. An increase in ET-1 with concomitant renal toxicity, secondary to VSP inhibition is referred to as “preeclampsia” [10] because both syndromes may have the same clinical features of hypertension, proteinuria and glomerular endotheliosis.

VEGF provides a survival signal to endothelial cells and in cancer xenograft models, endothelial cell loss within tumors is observed within days after initiation of antiangiogenic therapy. VEGF promotes endothelial cell survival and, conversely, inhibition of VEGF leads to endothelial cell apoptosis and chronic remodeling of the capillary beds, a process referred to as capillary rarefaction. Consequently, rarefaction, the presence of a diminished number of microvessels, has frequently been proposed as a mechanism of VSP inhibitor-associated hypertension [11]. Human studies demonstrate a significant decrease in dermal capillary density and decreased capillary dilatory response after VSP inhibitor treatment, implicating functional as well as anatomic attenuation of vessel density [12]. The role of rarefaction in the development or maintenance of hypertension remains questionable [13].

Activation of the renin-angiotensin-aldosterone (RAAS) pathway secondary to VSP inhibition is plausible biologically. Endothelial dysfunction caused by VSP inhibition might be expected to cause glomerular ischemia and upregulation of RAAS but in fact experimental evidence is not supportive of significant role of RAAS in mediating blood pressure elevation with VEGF inhibition. Renin and aldosterone levels did not increase after treatment with sorafenib [14]. Renin messenger RNA and aldosterone urinary excretion were actually decreased in DC101 (VEGF inhibitor) treated mice when compared with controls [8]. This view is challenged by the experimental evidence from mice treated with an anti-murine VEGF-A monoclonal antibody for 5 weeks [15]. In this study the inhibition of angiotensin-converting enzyme (ACE) by ramipril almost entirely prevented the adverse hemodynamic effects and LV remodeling in anti-VEGF–treated mice.

The renal effects of VEGF inhibition such as thrombotic microangiopathy or renovascular dysregulation may also contribute to hypertension [16].

Oxidative stress (increased bioavailability of reactive oxygen species) might also contribute to the development of hypertension during antiangiogenic therapy via oxidation of NO, thereby decreasing NO-mediated vasodilator tone [17].

Alterations in pressure-natriuresis relationship caused by VSP inhibition may explain the development of hypertension. Decreased levels of NO cause sodium retention and extracellular volume increase resulting in perpetuation of hypertension by changing the set-point for sodium excretion [18].

Recent observational study elegantly demonstrates that large artery properties are affected by VSP inhibition by sunitinib or sorafenib. These drugs cause the increase in arterial stiffness and this increase is partially independent of the blood pressure change [19].

Despite the lack of a comprehensive model, new-onset hypertension seems to be an on-target, intended effect of VEGF-pathway blockade during treatment with VSP inhibitors. On-target refers to exaggerated and adverse pharmacologic effects of VSP inhibitors at the target of interest (i.e. VEGF pathway). This concept is further supported by evidence that bevacizumab, a monoclonal anti-VEGF-A antibody, and aflibercept, a soluble receptor with affinity to VEGF-A and -B, are also strong inducers of hypertension [20, 21]. Inhibition of the VEGF pathway induces hypertension as a class effect; VEGFR-2 is most prominent of those [22–24].

In the majority of cases, proteinuria and hypertension resolve or significantly improve with removal of anti-VEGF therapy. There have been reports, however, of resolution of nephrotic range proteinuria after cessation of treatment, but with limited recovery of actual renal function [25].

### Incidence and timing of new –onset hypertension

New-onset hypertension may occur in cancer patients, regardless of treatment type or even in those not receiving treatment. The reported incidence depends on many factors including the different classification systems used by oncology and cardiac societies. In 2010 the National Cancer Institute updated Common Terminology Criteria for Adverse Events (CTCAE version 4.03) [26] in order to reflect more closely the Seventh Report of the Joint National Committee of Prevention, Detection, Evaluation, and Treatment of High Blood Pressure (JNC7) guidelines for hypertension. Specifically, the definition of hypertension and goals of treatment vary among different professional societies [27–30]. It should be noted that although the terminology defining hypertension remained relatively static, the numeric values used to further classify hypertension severity has changed over this timespan, as described in Table 1.

**Table 1:** Different definitions of hypertension

Definition of hypertension^a^							
Classification systems	CTCAE 3.0 (2006) (30)	CTCAE 4.03 (2010) (26)		JNC-7 (2003) (28)^b^		ESH/ESC (2013) (27)	
		Systolic	Diastolic	Systolic	Diastolic	Systolic	Diastolic
Grade 1	Asymptomatic Transient (<24 h) Increase >20 (diastolic) > 150/100 if previously normal	120–139	80–89	140–159	90–99	140–159	90–99
Grade 2	Recurrent/persistent (≥24 h) symptomatic increase >20 (diastolic) > 150/100 if previously normal	140–159	90–99	≥160	≥100	160–179	100–109
Grade 3	Requiring >1 drug Requiring more intensive therapy than previously	≥160	≥100	ND	ND	≥180	≥110
Grade 4	Life-threatening consequences	Life-threatening consequences	ND	ND	ND	ND	ND
Grade 5	ND	Death	ND	ND	ND	ND	ND

*Abbreviations: CTCAE* common terminology criteria for adverse events, *JNC* joint national committee, *ESH* European society of hypertension, *ESC* European society of cardiology, *ND* not defined

^a^ All numeric blood pressure values are in mm Hg

^b^ The current JNC-8 report (2013) [115] does not classify grades of hypertension

There is a great variability between the different VSP inhibitors in terms of the number of trials published, the variety of study populations and their sizes. Many publications include only reports of common (>10 % of cases) adverse events potentially underestimating the true incidence of hypertension [31, 32].

Most of treatment-induced hypertension are reported to be low-grade, and manageable with antihypertensive medications. Reports of hypertension requiring treatment cessation are scarce. The reported incidence of all-grade hypertension ranges from 28 % in the initial bevacizumab trials [33] with similar frequency for sorafenib [34], sunitinib [35], and vandetanib [36] and reaching about 40 % with pazopanib [37] and axitinib [38]. Hypertension incidence is higher with more potent VSP inhibitor therapies probably representing on-target effect. Of all VSP inhibitors axitinib, regorafenib and lenvatinib have the highest reported rates of all-grade and high-grade (grade ≥ 3) treatment-induced hypertension (Table 2).

**Table 2:** Relative risk of all-grade and high-grade (grade ≥ 3) hypertension of several VSP inhibitor agents, described in meta-analyses

	All grade hypertension			High grade hypertension		
	RR^a^	95 % CI	*p* Value	RR^a^	95 % CI	*p* Value
Bevacizumab [33]	3.02	2.24–4.07	<0.001	5.28	4.15–6.71	0.001
Sunitinib [52]	3.44	0.62–19.15	0.16	22.72	4.48–115.29	<0.001
Axitinib [38]	3.00	1.29–6.97	0.01	1.71	1.21–2.43	0.003
Sorafenib [62]	3.07	2.05–4.60	<0.01	3.31	2.21–4.95	<0.01
Pazopanib [65]	4.97	3.38–7.30	<0.001	2.87	1.16–7.11	0.023
Lenvatinib [69]	7.44	4.31–12.85	<0.001	18.2	5.90–56.32	<0.001
Vandetanib [36]	5.10	3.76–6.92	<0.001	8.06	3.41–19.04	0.001
Regorafenib [74]	3.76	2.35–5.99	<0.001	8.39	3.10–22.71	0.001

*Abbreviations: RR* relative risk, *CI* confidence interval

^a^ Relative risk of new onset hypertension during trial period in interventional group compared to control

Incidence of hypertension depends on drug variables (type of drug, dose and schedule used) and patients’ characteristics [39]. An absolute blood pressure increase occurs in the majority of patients, with rapid onset after the first administration of the drug. In the real-world registry of Partners Healthcare [40] the absolute observed mean increase has been described at 21 mmHg for systolic and 15 mmHg for diastolic blood pressure [40]. Risk factors included pre-existing hypertension (65.4 %), age > 60 years and body mass index (BMI) ≥25. Race, sex, the use of a specific anti-VEGF agent, and prior use of specific anti-hypertensive medications were not significant risk factors [40].

### Specific agents associated with the development of hypertension

#### Bevacizumab

Bevacizumab (Avastin®, Genentech/Roche) is a monoclonal antibody (mAb) that targets the VEGF pathway [41]. Most clinical trials compare combination therapy with bevacizumab and standard chemotherapy versus standard chemotherapy alone.

The incidence rate of all-grade and high-grade hypertension associated with bevacizumab is 23.6 % (RR 3.02, 95 % CI 2.24–4.07, *p* < 0.001) and 7.9 % (RR 5.28, 95 % CI 4.15–6.71, *p* < 0.001), respectively [42]. The malignant mesothelioma trial reported the highest incidence of high-grade hypertension (22.0 %), and the lowest rates were documented in pancreatic cancer (5.5 %). Unlike disease-specific effects seen in small-molecule TKIs that are VSP inhibitors, patients suffering from RCC had a 7.1 % incidence rate of high-grade hypertension, which was slightly lower than the overall rate of 7.9 % seen in patients with other malignancies [42]. While high-dose bevacizumab clearly seems to be linked with treatment-induced hypertension, the significance for low-dose regimens remains unsettled [42, 43]. Treatment-induced hypertension has not been shown to be a class effect in other mAbs that target the VEGF signaling pathway [44].

#### Sunitinib

Sunitinib malate (Sutent®, SU11248, Pfizer) is multi-targeted tyrosine kinase inhibitor (TKI) with inhibitory effects on multiple tyrosine kinase receptors, including VEGFR 1, 2, and 3, platelet-derived growth factor receptors (PDGFR) a and b, FMS (Feline McDonough Sarcoma)-like tyrosine kinase 3 (Flt-3), c-Kit, and RET receptor tyrosine kinase [45–49]. Treatment induced hypertension has been associated with sunitinib therapy for different forms of cancer. In the meta-analysis of early clinical trials including 4999 patients receiving sunitinib, the incidence of all-grade and high-grade hypertensions were 21.6 % (95 % CI: 18.7–24.8 %) and 6.8 % (95 % CI: 5.3–8.8 %) respectively. The risk may vary with tumor type and the dosing schedule of sunitinib. Sunitinib was associated with a significantly increased risk of high-grade hypertension (RR = 22.72, 95 % CI: 4.48 to 115.29, *p* < 0.001) and renal dysfunction (RR: 1.36, 95 % CI: 1.20 to 1.54, *p* < 0.001) in comparison with controls. Further subgroup analysis revealed a significant risk of hypertension for patients with RCC (25.9 % versus 20.4 %, RR 1.27, 95 % CI: 1.13–1.43 %, *p*  <  0.001). One possible explanation is that patients with RCC may have higher VEGF level than non-RCC patients, and the resulting overall anti-VEGF effect of sunitinib may be more evident. Alternatively, patients with RCC may have reduced renal function due to prior nephrectomies, and thus may have reduced excretion of sunitinib level leading to increased sunitinib exposure or directly contribute to the development of hypertension. Indeed, majority of patients with RCC in these trials had nephrectomies before receiving sunitinib [35].

More recent phase II trials have shown a significant risk of treatment-induced hypertension with sunitinib in patients suffering from pancreatic neuroendocrine tumors [50] and endometrial carcinoma [51]. Treatment-related hypertension incidence varied with the dose of sunitinib in a phase II study of women with epithelial endometrial and primary peritoneal cancers [52, 53].

#### Axitinib

Axitinib (Inlyta®, AG-013736, Pfizer) is a selective tyrosine-kinase inhibitor of VEGFR 1, 2, and 3 [54]. A meta-analysis of two phase III and eight phase II trials including 1148 patients showed high incidence rates for all-grade and high-grade hypertension, 40.1 and 13.1 % respectively. Compared with placebo, treatment with axitinib was associated with a significant risk for all-grade (RR 3.00, 95 % CI 1.29 to 6.97, *p* = 0.011) and high-grade hypertension (RR 1.71, 95 % CI 1.21 to 2.43, *p* = 0.003) [38]. Similar to the trend discussed regarding subgroup differences between treatment-induced hypertension in RCC and non-RCC patients, axitinib treatment had a significantly higher risk for all-grade hypertension (RR 1.69, 95 % CI 1.45 to 1.97, *p* < 0.001) and high-grade hypertension (RR 3.20, 95 % CI 2.30 to 4.46, *p* < 0.001) [38]. We further analyzed the data in the three non-RCC trials, and found that, in contrast to sunitinib, the risk of all-grade (RR 3.98, 95 % CI 2.68 to 5.89, *p* < 0.001) and high-grade hypertension was significant (RR 4.75, 95 % CI 1.86 to 12.18, *p* < 0.001) [55–57]. An additional meta-analysis that focused on patients with pancreatic malignancies also yielded similar results [58].

Rini et al. have published their results of 53 patients after 5-year follow-up and showed an even higher incidence rate of all-grade hypertension of 63.5 %. The incidence rate of high-grade hypertension incidence was 13.5 %, similar to that shown in short-term studies [59].

The incidence rates of treatment-induced hypertension associated with axitinib, are higher than those described for all multi-targeted TKIs discussed in this review. This contrasts with the evidence that multi-targeted agents are more prone to this effect. We challenge this principal, further suggesting alternative explanations for these observations: 1) axitinib might have more undiscovered targets of action; 2) the selective nature of axitinib encouraged the use of higher dosages, thus increasing efficacy and associated toxicity of the anti-VEGFR effect.

#### Sorafenib

Sorafenib (Nexavar®, BAY 43–9006, Bayer) is a multi-targeted TKI, which effects Raf kinase, VEGFR 1, 2, and 3, PDGFR-b, FLT-3, c-KIT and RET-receptor tyrosine kinase [60]. Treatment-induced hypertension with sorafenib is documented in different forms of cancer.

In three meta-analysis studies of sorafenib-associated hypertension, the incidence rates of all-grade and high-grade hypertension were 19.1–23.4 and 4.3–6.0 % [61, 62] respectively. The three studies showed similar relative risks; in the largest metaanalysis of 14 randomized controlled trials and 39 prospective single-arm trials involving 13,555 patients the relative risks of all-grade and high-grade hypertension were 3.07 (95 % CI 2.05 to 4.60, *p* < 0.01) and 3.31 (95 % CI 2.21 to 4.95, *p* < 0.01), respectively [61]. A subgroup analysis showed patterns similar to those described for other VEGFR inhibitors, with a significantly higher risk in patients suffering from RCC. In contrast to sunitinib, the incidence of treatment–associated hypertension with sorafenib was increased in patients with non-RCC, hepatocellular carcinoma (HCC), and non-small cell lung cancer (NSCLC). There was no associated risk of hypertension for patients with pancreatic cancer, breast cancer or acute myeloid leukemia. In patients with melanoma, only the risk of developing high-grade hypertension was significant [61].

Concomitant treatment with sorafenib and chemotherapy attenuated treatment-induced hypertension, without the need to lower the dose of sorafenib [61, 62].

#### Pazopanib

Pazopanib (Votrient®, GW786034, Novartis), is a multi-targeted TKI, targeting VEGFR 1, 2, and 3, PDGFR-a and PDGFR-b, and c-kit [63, 64]. A large meta-analysis found the incidence of all-grade and high-grade treatment-induced hypertension of 35.9 and 6.5 %, respectively. RCC patients had the highest incidence rates, however non-RCC patients also had a significant risk of developing hypertension [37].

In RCC patients pazopanib is associated with a higher risk of all-grade treatment-induced hypertension compared to sorafenib (RR 1.99, 95 % CI 1.73 to 2.29, *p* = 0.001) and sunitinib (RR 2.20, 95 % CI 1.92 to 2.52, *p* = 0.001). There was no significant difference regarding the incidence of high-grade hypertension between these three agents [37].

Although TKIs targeting the VEGFR pathway are more prone to causing hypertension in RCC patients, pazopanib has the highest documented rate of treatment-induced hypertension in patients with thyroid carcinoma, with all-grade hypertension occurring in 54.1 % of cases [65], versus 38.2 % in RCC patients [66, 67]. We have found that patients with metastatic thyroid carcinoma have a significant risk of developing hypertension, even when compared with RCC patients (RR 1.95, 95 % CI 1.42 to 2.69, *p* < 0.0001). This is in contrast to the trend of higher rates of treatment-induced hypertension seen with other VSP inhibitors discussed in this review.

#### Lenvatinib

(Lenvima®, E7080, Eisai) is an oral, multi-targeted tyrosine kinase inhibitor of the VEGFRs 1, 2, and 3, fibroblast growth factor receptor (FGFR) 1 through 4, PDGFR α, RET, and KIT signaling networks [68]. The drug was recently approved by the U.S. Food and Drug Administration [69] for the treatment of Iodine-131-refractory thyroid cancer [69]. Any grade hypertension was observed in 69.3 % of the patients in the lenvatinib group compared to 42.9 % in the placebo group and hypertension grade ≥3 was found in 9.2 and 2.3 % of the levatinib and placebo groups, respectively. Despite the high rate of new-onset hypertension, discontinuation of the drug was necessary in 1.1 % of patients only and dose reduction/interruption occurred in 19.9 % of the drug group.

#### Vandetanib

Vandetanib (Caprelsa®, ZD6474, Astra-Zeneca) targets VEGFR 2, VEGFR 3, and RET tyrosine kinase receptors. It has a negligible and week effect on VEGFR 1 [70]. This drug is not indicated for RCC as most of other agents previously reviewed. A meta-analysis including 1414 cases from 11 trials found all-grade and high-grade treatment-induced hypertension in an overall cancer population of 24.2 and 6.4 %, respectively. Both all-grade and high-grade hypertension rates were higher in medullary thyroid cancer (MTC) patients - 32.1 and 8.8 %, respectively. NSCLC patients also had a slightly higher incidence then the other patient groups (21.8 and 7.6 % versus 15.4 and 3.4 %, respectively). Overall there was a significant risk of treatment-induced hypertension, which according to a subgroup analysis varied between the MTC, NSCLC and non-MTC/NSCLC groups in line with the corresponding incidence rates [36].

#### Regorafenib

Regorafenib (Stivarga®, BAY 73–4506, Bayer) is a multi-targeted TKI that broadly targets VEGFR 1, 2, and 3, TIE-2, Ret-receptor, PDGF-b, basic fibroblast growth factor receptor-1, c-KIT, RAF-1, BRAF and p38 MAP kinase [71, 72]. A meta-analysis of 750 patients from five trials found a pooled incidence rate of all-grade and high-grade hypertension of 44.4 and 12.5 %, respectively. Treatment-induced hypertension rates were notable in RCC (49.0 %) and HCC (36.1 %) patients, similarly to other TKIs. Regorafenib-induced hypertension was seen in 56.1 and 27.8 % of patients with gastrointestinal stromal tumors (GIST) and metastatic colorectal cancer (mCRC), respectively. The risk for all-grade (RR 3.76, 95 % CI 2.35 to 5.99, *p* < 0.001) and high-grade (RR 8.39, 95 % CI 3.10 to 22.71, *p* < 0.001) hypertension associated with regorafenib therapy was increased significantly [73].

#### Cediranib

Cediranib (Recentin®, AZD2171, Astra-Zeneca), a relatively new agent, targets VEGFR 1, 2, and 3, and c-KIT [74–76].

A meta-analysis of four phase II and phase III trials of cediranib indicated increased risk of all-grade hypertension in 42.1 % of cases (RR 2.63, 95 % CI 1.61 to 4.29, *p* < 0.001) [23]. No data on high-grade hypertension was available in this study. Within clinical trials evaluating cediranib therapy there is respective evidence of treatment-induced all-grade and high-grade hypertension in patients with RCC (64–73 and 19–36 %) [77, 78], GIST (79 and 29 %) [80], soft tissue sarcoma (40 and 10 %) [79], HCC (41 and 21–29 %) [80, 81], small-cell lung cancer (52 and 12 %) [82], mesothelioma (70 and 22–32 %) [83, 84], and breast cancer (55 and 19 %) [85]. In a phase II trial of patients with ovarian cancer, combined treatment with cediranib and olaparib showed incidence rates of all-grade and high-grade hypertension of 80 and 41 %, respectively. In those patients only treated with olaparib, the incidence was zero [86]. We calculated the relative risks of treatment-induced hypertension according to published results of a phase III trial comparing cediranib and bevacizumab, both combined with mFOLFOX6. The relative risks for all-grade and high-grade hypertension were 1.60 (95 % CI 1.38 to 1.87, *p* < 0.0001) and 1.69 (95 % CI 1.08 to 2.64, *p* = 0.02) respectively [87].

#### Other notable VSP inhibitors

We found reports of treatment-induced hypertension caused by other TKIs, regarding cabozantinib [88, 89], nintedanib [90, 91] and ponatinib [92], inhibitors of the VEGFR pathway.

Among small-molecule TKIs, some agents induce hypertension regardless of VEGF inhibition. These include trametinib, a MEK1/MEK2 inhibitor that does not appear to affect the VEGF pathway [93, 94]. Interestingly, this effect was shown in patients with NSCLC, similar to TKIs that affect the VEGF pathway, and is not described in melanoma and pancreatic cancer [95–97]. Another non-VSP inhibitor TKI shown to induce hypertension is ibrutinib, a Bruton’s tyrosine kinase inhibitor, used for treatment of various hematologic malignancies [98].

### Clinical significance of hypertension in subsequent cardiac morbidity and as a possible biomarker

The inhibition of the VEGFR pathway may lead to further cardiovascular complications, as these pathways have an effect on the maintenance of myocardial homeostasis under various stressors [99–102]. Animal models show that cardiotoxicity may present itself secondary to hypertension [103], yet this hypothesis is not supported in humans [104].

Several studies have described correlations between treatment-induced hypertension and prognostic parameters such as progression-free survival (PFS), overall survival (OS), and overall response rate (ORR) [61, 105–111]. These studies propose that the effect may be dose-dependent and that hypertension might be indicative of effective VEGF inhibition and a positive antiangiogenic response, and as such could be a biomarker of a favorable outcome from VSP inhibitors treatment. In the recent analysis of 770 sunitinib-treated metastatic RCC (mRCC) patients on-treatment hypertension was associated with longer PFS (HR 0.37, 95 % CI 0.27–0.52, *P* < 0.001), and better OS (HR 0.36, 95 %CI 0.27–0.50, *P* < 0.001) [112]. Similar results were observed in the study of 38 hepatocellular carcinoma patients treated with sorafenib: time to progression was 153 days in the hypertension vs 50.5 days in the non-hypertension group (*P* < 0.017) and median OS was 1329 days in the hypertension group versus 302 days in the non-hypertension group (*P* < 0.004) [113]. In contrary, according to the retrospective analysis of 337 metastatic soft tissue sarcoma (STS) patients, pazopanib-induced hypertension did not correlate with outcome in pazopanib-treated STS patients. Duffaud et al. [114] conclude that the occurrence of hypertension cannot serve as biomarker in this setting. Probably, using hypertension as biomarker of VSP inhibitor treatment favorable outcomes depends on disease process and drug.

### Management of hypertension

In the review of previously described studies, treatment-induced hypertension secondary to VSP inhibition was most commonly low-grade, and easily corrected with standard antihypertensive medications. Traditional recommendations regarding lifestyle changes including physical exercise, weight reduction, dietary change and sodium restriction, though potentially beneficial, may be unachievable in the clinical setting of advanced malignancy [115]. Treatment goal of VSP inhibitor induced hypertension should be diminishing the short-term risk of stroke, myocardial infarction, heart failure while ensuring effective anticancer (antiangiogenic) therapy. Recent meta-analyses pointed to relative risk of 2.23 of fatal adverse events related to VSP inhibitors compared to controls [116]. In the absence of specific evidence-based clinical guidelines Cardiovascular Toxicities Panel of the National Cancer Institute recommended formal cardiovascular risk assessment before VSP inhibitor treatment, active monitoring of blood pressure and cardiac toxicity and aggressive management of blood pressure elevations and early signs and symptoms of cardiac toxicity to prevent clinically significant complications of therapy [117]. Blood pressure target for treatment is <140/90 mm Hg [118, 119]. Targeting the RAAS has been advocated although whether there is any significant difference between angiotensin converting enzyme inhibitors (ACEi) or angiotensin receptor antagonists (ARBs) remains inconclusive [120]. A recent study by McKay et al. showed a favorable outcomes in patient with RCC receiving either ACEi or ARBs [121]. The use of these drugs must take into account the possible impact on renal function and subsequent effects on drug metabolism during concomitant treatment with cytotoxic agents, which depend on renal clearance for their metabolism, such as cisplatin and pemetrexed. More recent reports cast doubt on the ability of ACEis and ARBs to have suffice effect in high grade hypertension [122]. Dihydropyridine calcium channel blockers may be preferable, specifically in elderly patients who suffer from isolated systolic hypertension [122]. It is also suggested that calcium channel blockers may beneficially counteract a reduction in NO production secondary to VSP inhibition, which would be expected to further enhance their vasodilatory mode of action [122]. However, only dihydropyridine calcium channel blockers, such as amlodipine or nifedipine, should be used, because nondihydropyridine calcium channel blockers, such as diltiazem or verapamil (inhibitors of cytochrome P450 3A4), which metabolizes VEGF inhibitors might lead to potentially high levels in plasma. Caution must be applied, while using diuretic therapy to avoid the risk of electrolyte imbalance. Beta-blocker therapy is a valuable option, especially in the presence of left ventricular dysfunction or arrhythmia [120]. When treating patients with antihypertensive agents, special attention needs to be paid to VSP inhibitor drug protocols in which there are “off” periods, during which there might be symptomatic rebound hypotension and risk of stroke.

## Conclusions

Treatment induced hypertension secondary to VSP inhibition therapy is a common on-target effect that rarely requires cessation of cancer therapy. Observed rates of hypertension may be challenging to quantify for individual drugs in part reflecting changes to treatment guidelines.

In our opinion, treatment-induced hypertension with VSP inhibitors will become more prevalent with increased use of these drugs reflecting earlier diagnosis of tumors and hence longer duration of therapy. We advise clinicians to be attentive to this eventuality and ensure that blood pressure is carefully monitored during a patient’s follow-up.

There are unanswered questions regarding the nature of the association between the VSP inhibition and the induction of hypertension, which may further clarify why some agents are more prone to this effect than others. Furthermore, it remains unclear why certain malignancies, such as RCC, are more susceptible to treatment-induced hypertension than other cancers.

The precise pathophysiology is still not fully understood. Future trials are also needed to determine the optimal choice of antihypertensive agents and therapeutic goals, which will take into account the complexity of the advanced oncologic patient, as well as more individual factors that may apply in specific types of cancer or other comorbidities.
